# Supplementary material for: The causal role of circulating inflammatory markers in osteoporosis: a bidirectional Mendelian randomized study
Source: Front Immunol. 2024 Jul 18;15:1412298. doi: 10.3389/fimmu.2024.1412298 (PMC11291241; doi:10.3389/fimmu.2024.1412298)
Supplement: Supplementary file 1 [file DataSheet_1.zip › supplementary material/CIM-OSTEOPOROSIS /GCST90274834.csv_leave_one_out.pdf]

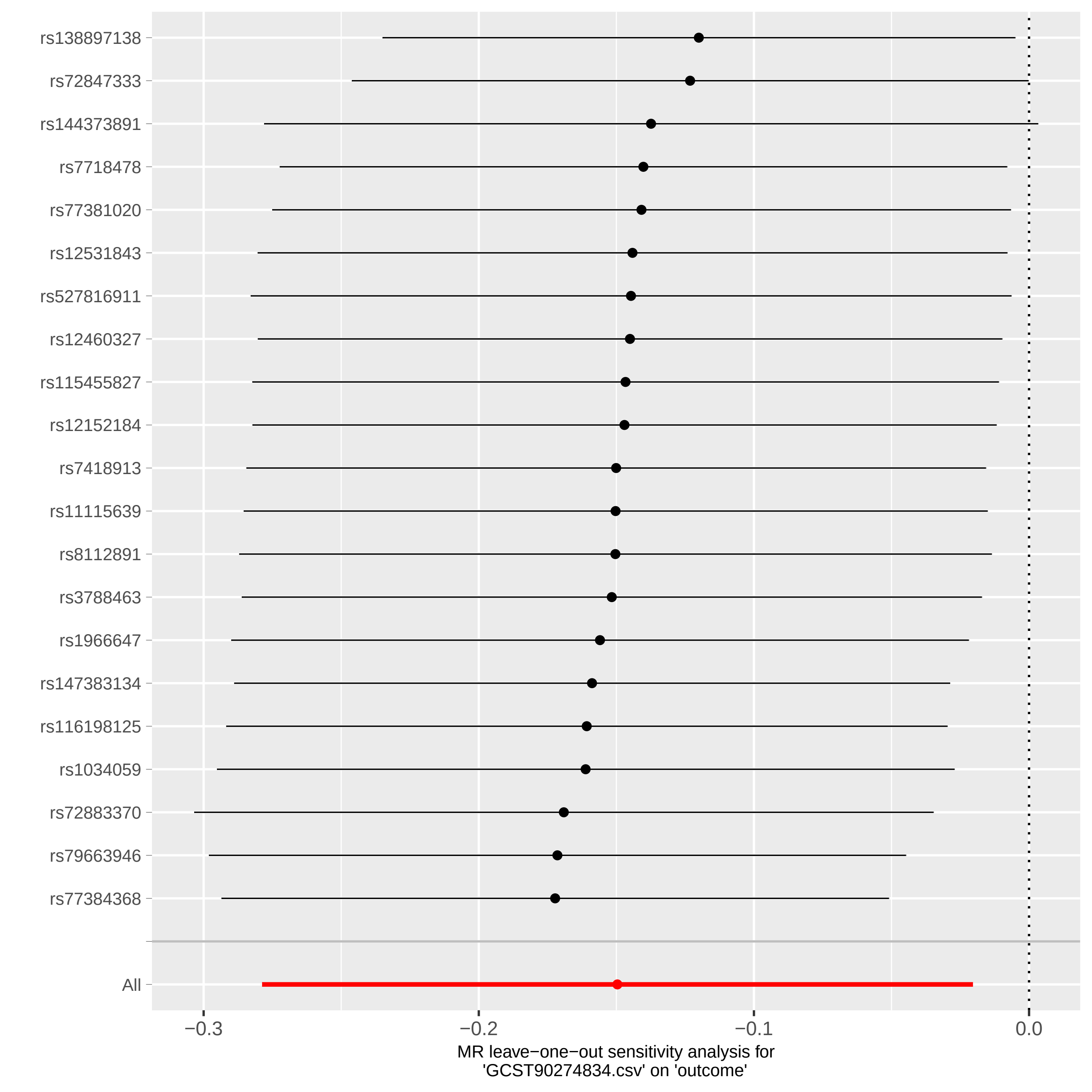
rs138897138
rs72847333
rs144373891
rs7718478
rs77381020
rs12531843
rs527816911
rs12460327
rs115455827
rs12152184
rs7418913
rs11115639
rs8112891
rs3788463
rs1966647
rs147383134
rs116198125
rs1034059
rs72883370
rs79663946
rs77384368
All
−0.3
−0.2
−0.1
0.0
MR leave−one−out sensitivity analysis for
'GCST90274834.csv' on 'outcome'
